# Prognostic value of nectin-4 in human cancers: A meta-analysis

**DOI:** 10.3389/fonc.2023.1081655

**Published:** 2023-03-03

**Authors:** Rongqiang Liu, Kailiang Zhao, Kunpeng Wang, Lilong Zhang, Wangbin Ma, Zhengdong Qiu, Weixing Wang

**Affiliations:** Department of Hepatobiliary Surgery, Renmin Hospital of Wuhan University, Wuhan, Hubei, China

**Keywords:** nectin-4, cancer, prognosis, meta-analysis, survival outcome

## Abstract

**Background:**

Many reports have described that abnormal nectin-4 expression may be used as a prognostic marker in many tumors. However, these studies failed to reach a consensus. Here, we performed a meta-analysis to comprehensively evaluate the prognostic value of nectin-4 in cancers.

**Methods:**

Relevant studies were identified through a comprehensive search of PubMed, EMBASE and Web of science until August 31, 2022. Pooled hazard ratios (HRs) with 95% confidence intervals (CIs) were used to evaluate the relationship between nectin-4 expression and overall survival (OS) and disease-free survival/progression-free survival/relapse-free survival (DFS/PFS/RFS). Odds ratios (ORs) with 95% CIs were applied to assess the relationship between nectin-4 expression and clinicopathologic features. Subgroup analysis was performed to explore the sources of heterogeneity. Sensitivity analysis and funnel plot were used to test the reliability of the results. All data analyses were performed using STATA version 12.0 software.

**Results:**

Fifteen articles involving 2245 patients were included in the meta-analysis. The pooled analysis showed that high nectin-4 expression was significantly associated with poor OS (HR: 1.75, 95% CI: 1.35–2.28). There was no relationship between high nectin-4 expression and DFS/PFS/RFS (HR: 178, 95% CI: 0.78–4.08).Subgroup analyses revealed that that high nectin-4 expression mainly presented adverse OS in esophageal cancer (EC) (HR: 1.78, 95% CI: 1.30–2.44) and gastric cancer (GC) (HR: 1.92, 95% CI: 1.43–2.58). We also found that high nectin-4 expression was associated with tumor diameter (big vs small) (OR: 1.96, 95% CI: 1.02–3.75), tumor stage (III-IV vs I-II) (OR: 2.04, 95% CI: 1.01–4.12) and invasion depth (T3+T4 vs T2+T1) (OR: 3.95, 95% CI: 2.06–7.57).

**Conclusions:**

Nectin-4 can be used as an effective prognostic indicator for specific cancers.

## Introduction

Cancer has surpassed all other diseases and become the leading cause of death worldwide. The morbidity and mortality of cancer have shown an upward trend and placed huge burdens on the medical system. There were 18.1 million new cancer cases and 9.6 million cancer deaths worldwide in 2018 (1). Infiltration and metastasis are vital biological behaviors that affect tumor progression. Cell adhesion molecules play crucial roles in this process. Understanding the mechanism of tumor invasion and metastasis can help to adopt appropriate strategies for timely prevention and treatment.

In recent years, the nectin family has been found to be involved in cell adhesion (2). Nectins belong to the immunoglobulin superfamily of Ca+ independent cellular adhesion molecules, and can bind to the actin cytoskeleton through a filamentous actin binding protein called afadin (2, 3). Nectin family members are involved in cell-cell connections and regulate cell activities including polarization, differentiation, migration, proliferation and survival (4).Nectin family members include nectin-1, -2, -3, and -4. Nectin-1, -2, and -3 are widely expressed in human tissues, while nectin-4 is mainly found in embryos and tumor tissues (5).

Nectin-4 known as poliovirus receptor-like protein (PVRL-4) can mediate virus entry into cells (6).The nectin-4 protein is transcribed from chromosome 6, and is a 66 KD cell adhesion molecule. Fabre-Lafay firstly found that nectin-4 was highly expressed in breast cancer (BC) tissue and was associated with invasion and metastasis of BC (7).Subsequently, it was confirmed that nectin-4 played an important role in adhesion, migration and proliferation in various tumor cells (8). Many studies found that abnormal expression of nectin-4 was closely related to the clinicopathological characteristics and prognosis of patients in a variety of tumors (9–23). However, the results of these studies were inconsistent.

The clinical value of nectin-4 as a potential tumor target in human tumors has not been fully described. Therefore, we performed a comprehensive meta-analysis to evaluate the prognostic role of nectin-4 in human tumors.

## Material and methods

### Search strategy

Three researchers(Rongqiang Liu, Kailiang Zhao and Kunpeng Wang) independently searched Pubmed, Embase and Web of science databases to find relevant articles about the prognostic significance of nectin-4 in human cancers. The search deadline was August 31, 2022. The following keywords were used: “Nectin-4” OR “PVRL4” OR “poliovirus-receptor-like 4” AND “cancer” OR “carcinoma” OR “neoplasm” OR “tumor” OR “tumour”. Language restriction was set in English. We manually searched the references included in the studies.

### Study selection

Articles were included in this meta-analysis if they met the following criteria: (1) investigated the association between nectin-4 expression and survival outcomes of cancer patients; (2) provided sufficient data to compute hazard ratios (HRs) or odds ratios (ORs) with 95% confidence intervals (CIs); (3) detected nectin-4 expression in human tumor tissue or serum; (4) articles published in English. The exclusion criteria were: (1) insufficient data to calculate HRs and 95% CIs; (2) case reports, conference papers, reviews, letters and articles published in non-English languages; (3) animal or cell experiments; (4) data from public databases.

### Data extraction and quality assessment

Three researchers (Rongqiang Liu, Kailiang Zhao and Kunpeng Wang) independently extracted the data from the included articles. Disagreement was settled by discussion. The following information was extracted: name of the first author, publication year, country, study type, tumor type, detected sample, detection method, survival analysis, analysis type and source of HRs. Multivariate analysis was adopted due to its higher accuracy. The quality of each study was assessed using the Newcastle–Ottawa Quality Assessment Scale (NOS) (24). NOS scores ranged from 0 to 9. NOS score from 0-4 was considered as low quality, 5-6 as medium quality, and 7-9 as high quality.

### Statistical analysis

HRs or ORs with corresponding 95% CIs were applied to analyze survival outcomes and clinicopathological features. We preferred to use the results of multivariate analysis. For literatures that could not directly extract survival data, we extracted survival results from survival plots according to Tierney et al. method (25).The forest plot was used to explore the prognostic role of nectin-4 in cancers. The p value <0.05 or I^2^ >50% was defined as significant heterogeneity, and the random-effects model was used to pooled HRs. Otherwise, the fixed-effect model was used. Sources of heterogeneity were explored through subgroup analysis. Sensitive analysis was applied to test the stability of the results. Begg’s test and Egger’s test were used to assess publication bias. P < 0.05 denoted statistical significance. STATA version 12.0 software (Stata Corporation, College Station, TX, USA) was used to perform all the statistical analysis.

## Results

### Search results

**Figure 1:**
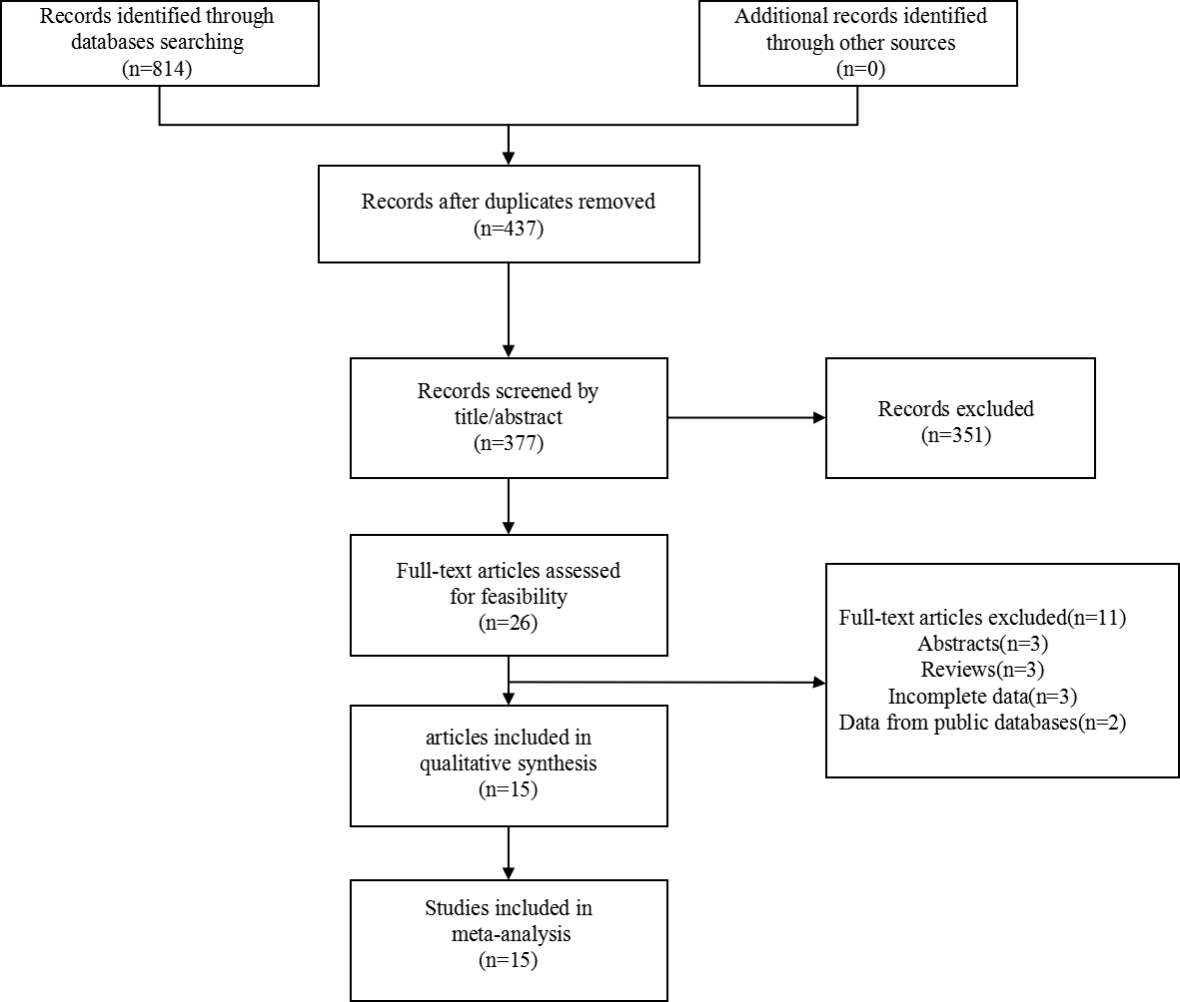
Flow chart of the article search.

Articles about the prognosis significance of nectin-4 in tumors were searched by retrieving the specified databases. We primarily identified 814 articles. After removal of 437 duplicate publications, 377 articles were remained. After browsing the title and abstract, 351 articles were excluded. 11 articles were further excluded after viewing the full text. Finally, we identified fifteen articles published from 2011 to 2020. The search flow diagram was displayed in **Figure 1**.

### Study characteristics

Fifteen articles containing 2245 patients were included in the meta-analysis. Ten articles were from Asia, and five were from the West. Fourteen articles included overall survival (OS) data. Four articles reported disease-free survival/progression-free survival/relapse-free survival (DFS/PFS/RFS)data. All articles detected nectin-4 expression in tumor tissues and used immunohistochemistry to identify nectin-4 expression. Nine studies directly provided the HRs and 95% CIs. The HRs and 95% CIs of the other articles were extracted from the survival curves. A total of seven different types of tumors were included, namely BC (9, 13, 15, 19, 23),esophageal cancer(EC) (10, 11, 18),hepatocellular carcinoma(HCC) (12),pancreatic cancer(PC) (14),lung cancer(LC) (16), upper tract urothelial carcinoma(UBC) (17),gastric cancer(GC) (20, 21) and gallbladder cancer(GBC) (22). The NOS scores of the included studies ranged from 6 to 7 (mean: 6.26). Basic information of the included studies was summarized in **Table 1**.

**Table 1:** Basic information of included articles.

Study	Year	Country	Study type	Tumor type	Sample size	Detected sample	Detected method	Survival analysis	Analysis type	Source of HR	NOS score
Athanassiadou	2011	Greece	Retrospective	BC	140	Tissue	IHC	OS	Univariate	SC	6
Deng	2019	China	Retrospective	EC	82	Tissue	IHC	OS	Multivariate	Reported	6
Lin	2019	China	Retrospective	EC	82	Tissue	IHC	OS	Multivariate	Reported	6
Ma	2016	China	Retrospective	HCC	87	Tissue	IHC	OS、RFS	Multivariate	Reported	7
M-Rabet	2016	France	Retrospective	BC	290	Tissue	IHC	OS	Multivariate	Reported	7
Nishiwada	2015	Japan	Retrospective	PC	123	Tissue	IHC	OS	Multivariate	Reported	6
Rajc	2017	Croatia	Retrospective	BC	147	Tissue	IHC	OS、DFS	Univariate	SC	7
Takano	2009	Japan	Retrospective	LC	422	Tissue	IHC	OS	Univariate	SC	6
Tomiyama	2020	Japan	Retrospective	UBC	99	Tissue	IHC	OS、PFS	Multivariate	Reported	7
Wang	2020	China	Retrospective	EC	84	Tissue	IHC	OS	Univariate	Reported	6
Zeindler	2019	Switzerland	Retrospective	BC	148	Tissue	IHC	OS	Multivariate	Reported	6
Zhang	2017	China	Retrospective	GC	64	Tissue	IHC	OS	Univariate	SC	6
Zhang	2018	China	Retrospective	GC	212	Tissue	IHC	OS	Multivariate	Reported	6
Lattanzio	2014	Italy	Retrospective	BC	197	Tissue	IHC	DFS	Multivariate	SC	6
Zhang	2016	China	Retrospective	GBC	68	Tissue	IHC	OS	Multivariate	Reported	6

BC, breast cancer; EC, esophageal cancer; HCC, hepatocellular carcinoma; PC, pancreatic cancer; LC, lung cancer; UBC, upper tract urothelial carcinoma; GC, gastric cancer; GBC, gallbladder cancer; OS, overall survival; DFS,disease-free survival; PFS, progression-free survival; RFS, relapse-free survival; SC, survival curve.

### Meta-analysis findings

#### Nectin-4 and OS

**Figure 2:**
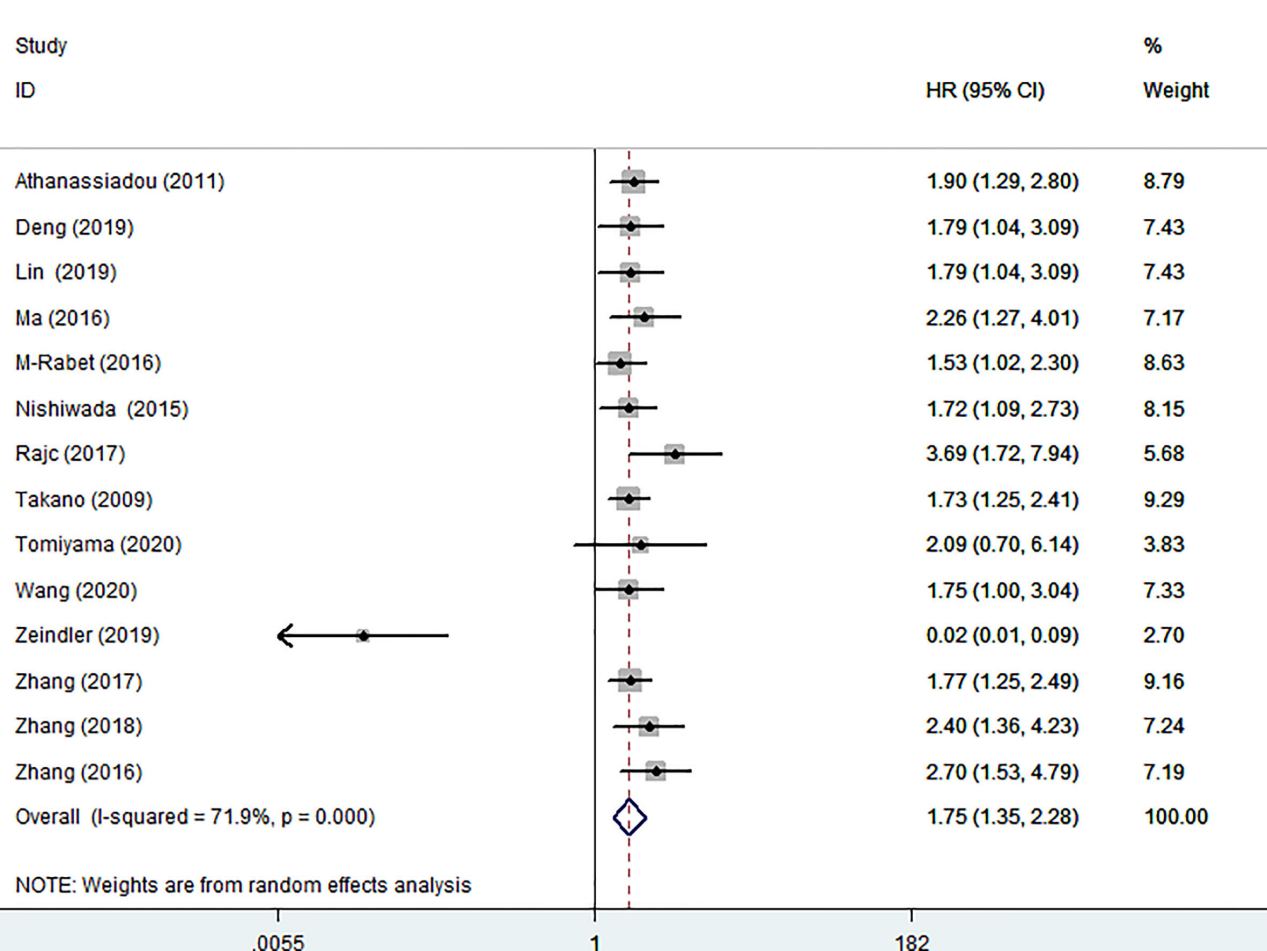
Forest plot of the relationship between high Nectin-4 expression and OS.

Fourteen articles involving 2048 patients displayed OS data. Due to obvious heterogeneity (I^2^ = 71.9%), a random model was used to calculate the pooled HRs with 95% CIs. The pooled results revealed that high nectin-4 expression was significantly associated with poorer OS (HR: 1.75, 95% CI: 1.35–2.28) (**Figure 2**).

#### Subgroup analysis for OS

**Figure 3:**
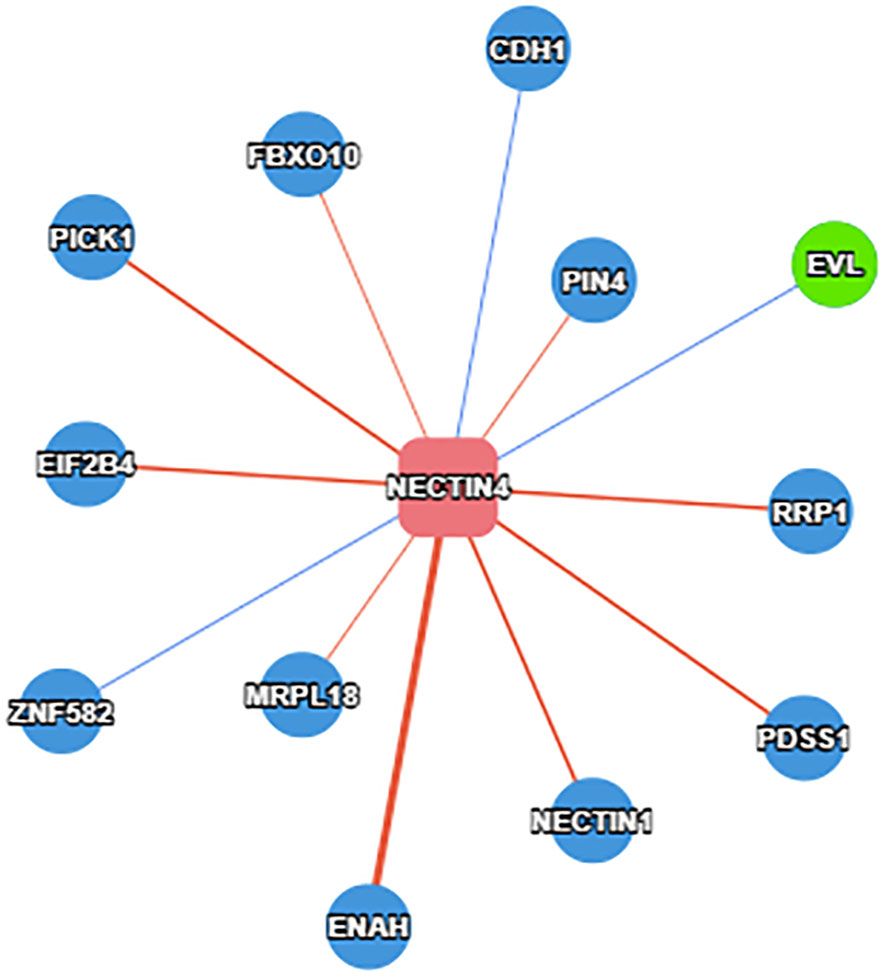
Protein that binds to the nectin-4 in BC.

In order to better detect the predictive effect of nectin-4, subgroup analysis was implemented based on the cancer type, analysis type, race, country, source of HRs and sample size (**Table 2**). We found that high nectin-4 expression was mainly associated with adverse OS in the subgroups of EC (HR: 1.78, 95% CI: 1.30–2.44), GC (HR: 1.92, 95% CI: 1.43–2.58), univariate analysis (HR: 1.86, 95% CI: 1.55–2.24), Asian (HR: 1.89, 95% CI: 1.62–2.21), China (HR: 1.98, 95% CI: 1.63–2.39), Japan (HR: 1.75, 95% CI: 1.35–2.26), reported (HR: 1.55, 95% CI: 1.03–2.33), SC (HR: 1.88, 95% CI: 1.54–2.28) and sample size<100 (HR: 1.93, 95% CI: 1.58–2.36). We also observed the absence of heterogeneity in some subgroups (I^2^ = 0), including cancer type (EC and GC), analysis type (univariate analysis), race (Asian), country (China and Japan) and sample size (< 100). These factors may be sources of heterogeneity. We further performed a subgroup analysis of BC. We found that nectin-4 expression was not associated with prognosis of patients with triple-negative BC. Due to the small numbers of included articles, more studies were needed to explore the relationship between nectin-4 expression and different subtypes of BC. In addition, we evaluated protein that bound to the nectin-4 in BC using the PINA database (26).We found that 12 proteins (PICK1,FBXO10,CDH1,PIN4,EIF2B4,EVL,ZNF582,MRPL18,ENAH,NECTN1,PDSS1 and RRP1) were associated with nectin-4 in BC (**Figure 3**).

**Table 2:** Subgroup analysis for OS.

Stratifed analysis	No. of studies	Pooled HR (95%CI)	P-value	Heterogeneity		
				I² (%)	P-value	Model
Cancer type						
BC HER2 negative Triple-negative Invasive	4121	0.88 (0.30-2.64) 3.69 (1.72-7.94) 0.2 (0.003-12.36) 1.9 (1.29-2.80)	0.824 0.439	92.8 97	0 0	Random
EC	3	1.78 (1.30-2.44)	0	0	0.997	Fixed
GC	2	1.92 (1.43-2.58)	0	0	0.366	Fixed
Others	5	1.93 (1.55-2.40)	0	0	0.679	Fixed
Analysis type						
Univariate analysis	5	1.86 (1.55-2.24)	0	0	0.492	Fixed
Multivariate analysis	9	1.50 (0.95-2.38)	0.081	81.3	0	Random
Race						
Caucasian	4	0.88 (0.30-2.64)	0.824	92.8	0	Random
Asian	10	1.89 (1.62-2.21)	0	0	0.950	Fixed
Country						
China	7	1.98 (1.63-2.39)	0	0	0.852	Fixed
Japan	3	1.75 (1.35-2.26)	0	0	0.945	Fixed
Source of HR						
Reported	10	1.55 (1.03-2.33)	0.035	78.9	0	Random
SC	4	1.88 (1.54-2.28)	0	10.5	0.34	Fixed
Sample size						
≥100	7	1.42 (0.85-2.37)	0.177	86.2	0	Random
<100	7	1.93 (1.58-2.36)	0	0	0.906	Fixed

BC, breast cancer; EC, esophageal cancer; GC, gastric cancer; SC, survival curve.

#### Nectin-4 and DFS/PFS/RFS

**Figure 4:**
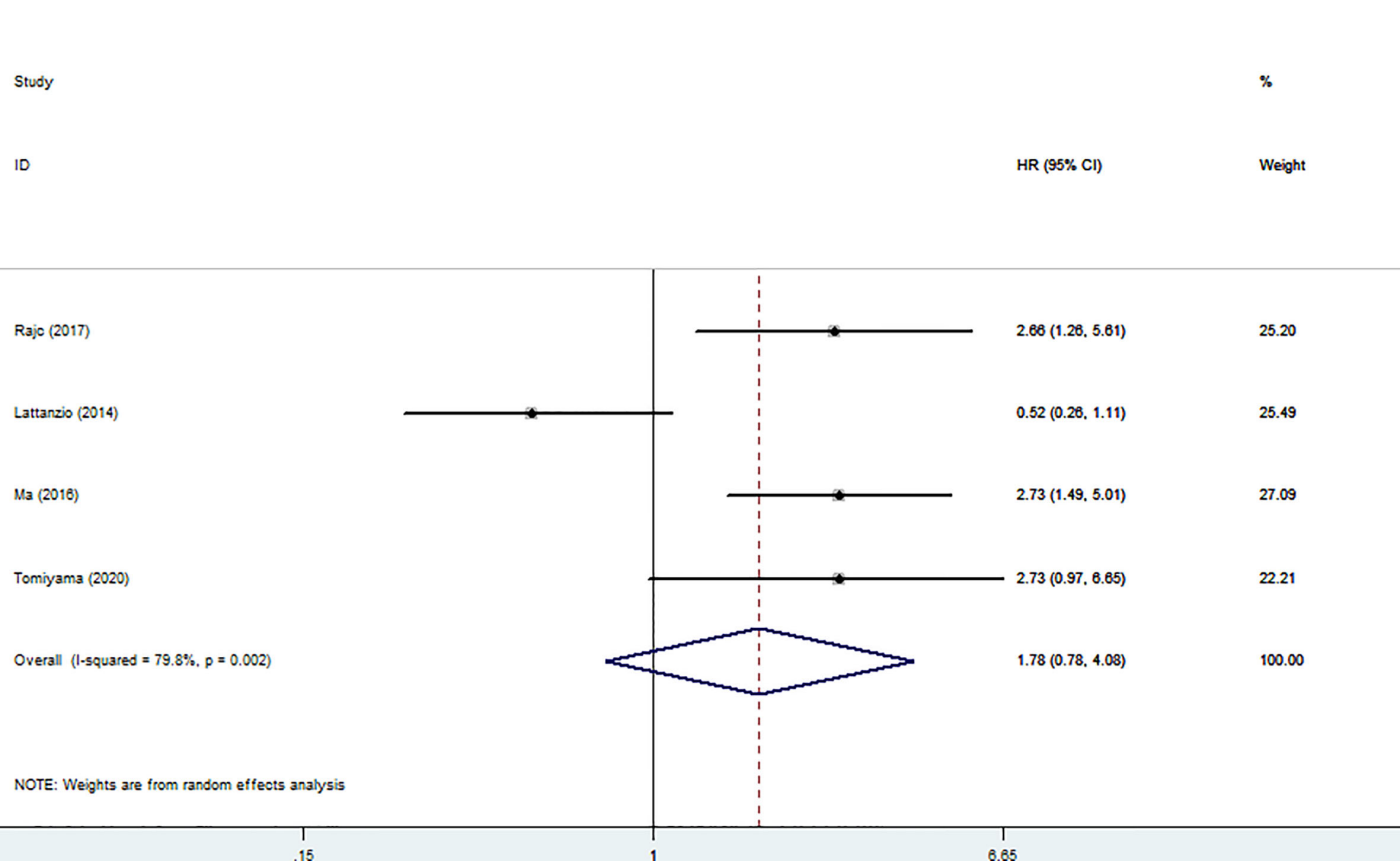
Forest plot of the relationship between high Nectin-4 expression and DFS/PFS/RFS.

Four articles involving 530 patients reported DFS/PFS/RFS data. We used a random model to calculate pooled HRs with 95% CIs because of the obvious heterogeneity (I^2^ = 79.8%). The results showed no relationship between high nectin-4 expression and DFS/PFS/RFS (HR: 178, 95% CI: 0.78–4.08) (**Figure 4**).

#### Nectin-4 and clinicopathological features

To investigate the relationship between high nectin-4 expression and clinicopathological features, we collected all data about high nectin-4 expression and clinicopathological features, such as sex, age, tumor diameter, tumor stage, tumor differentiation, lymph node status, distant metastasis and invasion depth metastasis (**Table 3**). The pooled results revealed that high Nectin-4 expression was associated with tumor diameter (big vs small) (OR: 1.96, 95% CI: 1.02–3.75), tumor stage (III-IV vs I-II) (OR: 2.04, 95% CI: 1.01–4.12) and invasion depth (T3+T4 vs T2+T1) (OR: 3.95, 95% CI: 2.06–7.57). Additionally, we noted that there was no significant relationship between high nectin-4 expression and gender (male vs female), age (old vs young), tumor differentiation (poor vs moderate/well), lymph node status (yes vs no) and distant metastasis (yes vs no).

**Table 3:** Association between high Nectin-4 expression and clinicopathological features.

Clinicopathologic features	No. of studies	Estimate OR (95%CI)	p-value	Heterogeneity		
				I2(%)	p-value	Model
Gender (male vs female)	10	0.96(0.75-1.23)	0.759	0	0.721	Fixed
Age (old vs young)	9	1.05(0.82-1.33)	0.704	0	0.999	Fixed
Tumor diamter (big vs small)	9	1.96(1.02-3.75)	0.043	71.5	0	Random
Tumor stage ((III-IV vs I-II)	8	2.04(1.01-4.12)	0.047	71.6	0.001	Random
Tumor differentiation (poor vs moderate/well)	2	0.97(0.139-6.74)	0.975	87.3	0.003	Random
Lymph node status (yes vs no)	9	1.77(0.51-6.21)	0.371	94	0	Random
Distant metastasis(yes vs no)	2	0.50(0.24-1.05)	0.066	37.4	0.206	Random
Invasion depth(T3+T4 vs T2+T1)	3	3.95(2.06-7.57)	0	0	0.97	Fixed

### Sensitivity analysis

**Figure 5:**
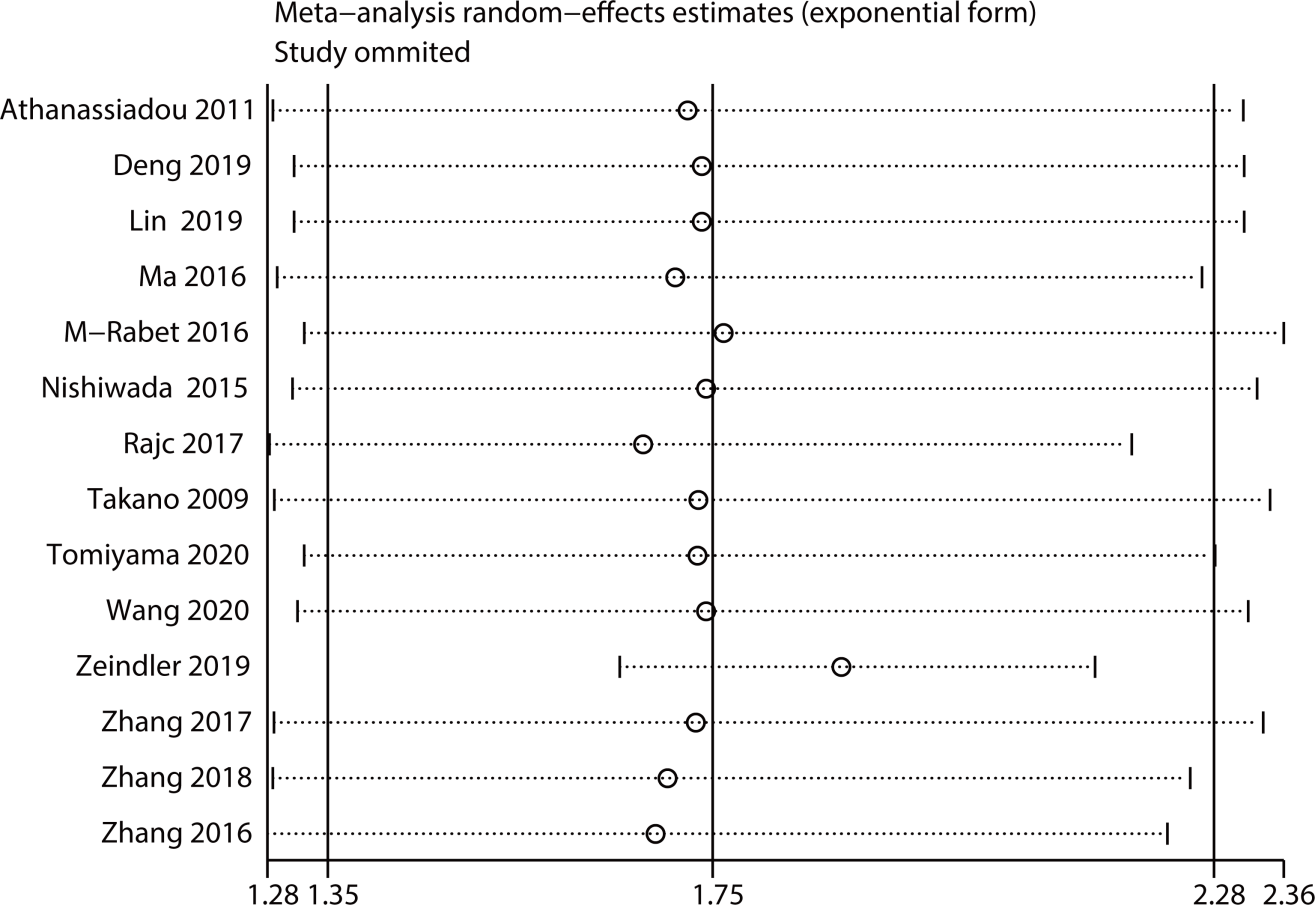
Sensitivity analysis for OS.

**Figure 6:**
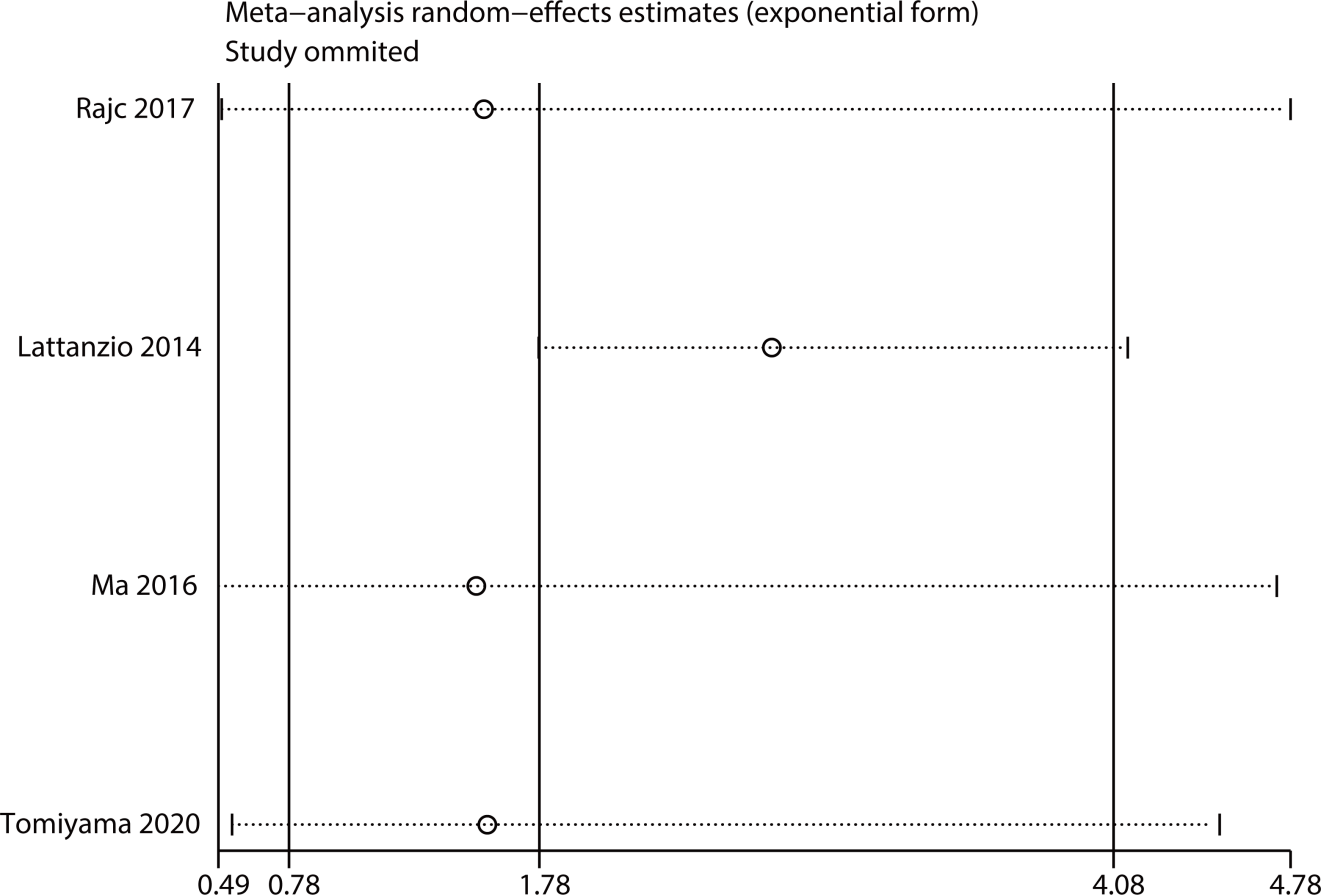
Sensitivity analysis for DFS/PFS/RFS.

Sensitivity analysis was performed by removing one study at a time. The results were consistent with the comprehensive analysis, confirming that the outcomes were stable (**Figures 5**, **6**).

### Publication bias

**Figure 7:**
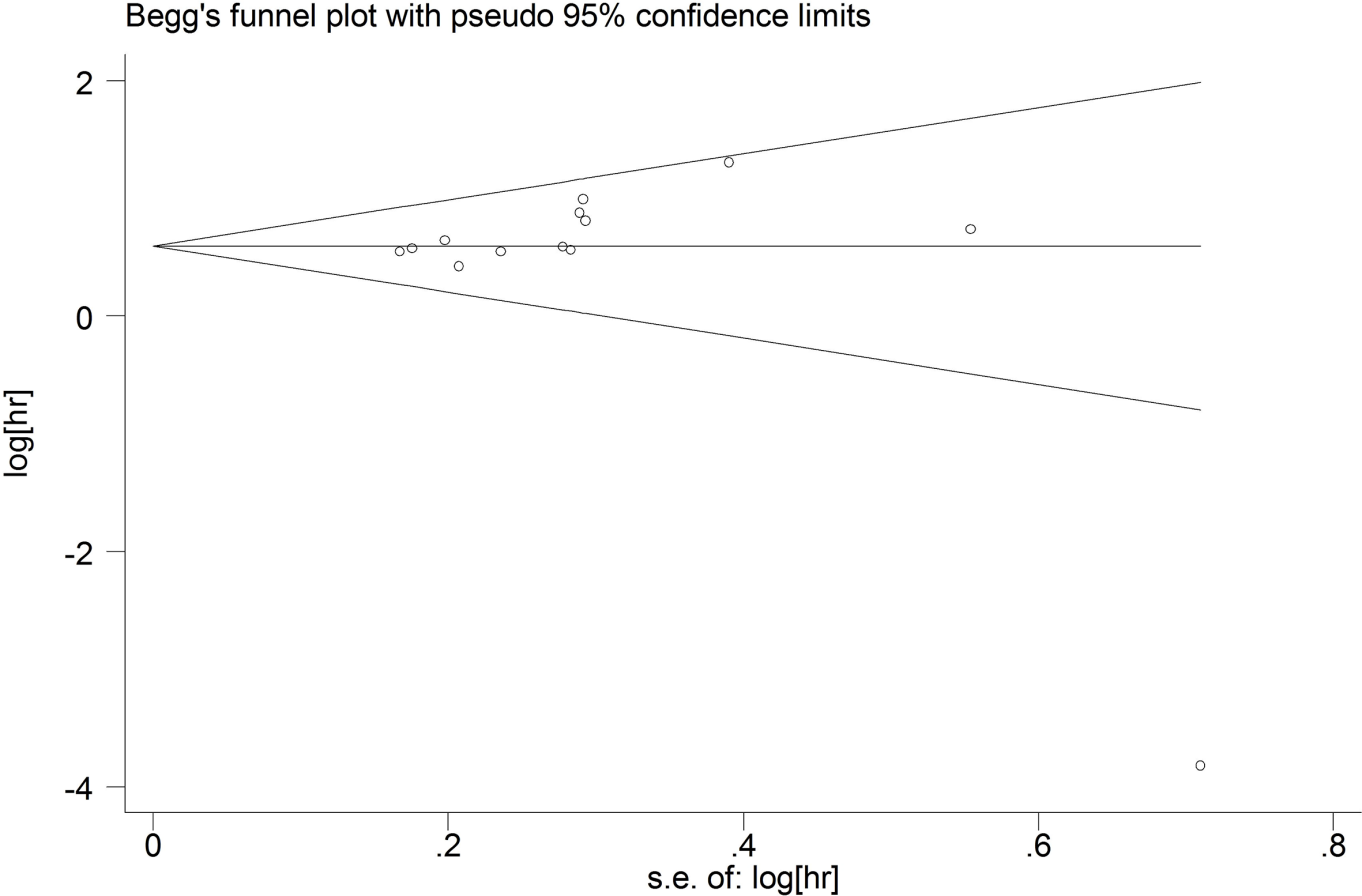
The publication bias for OS.

**Figure 8:**
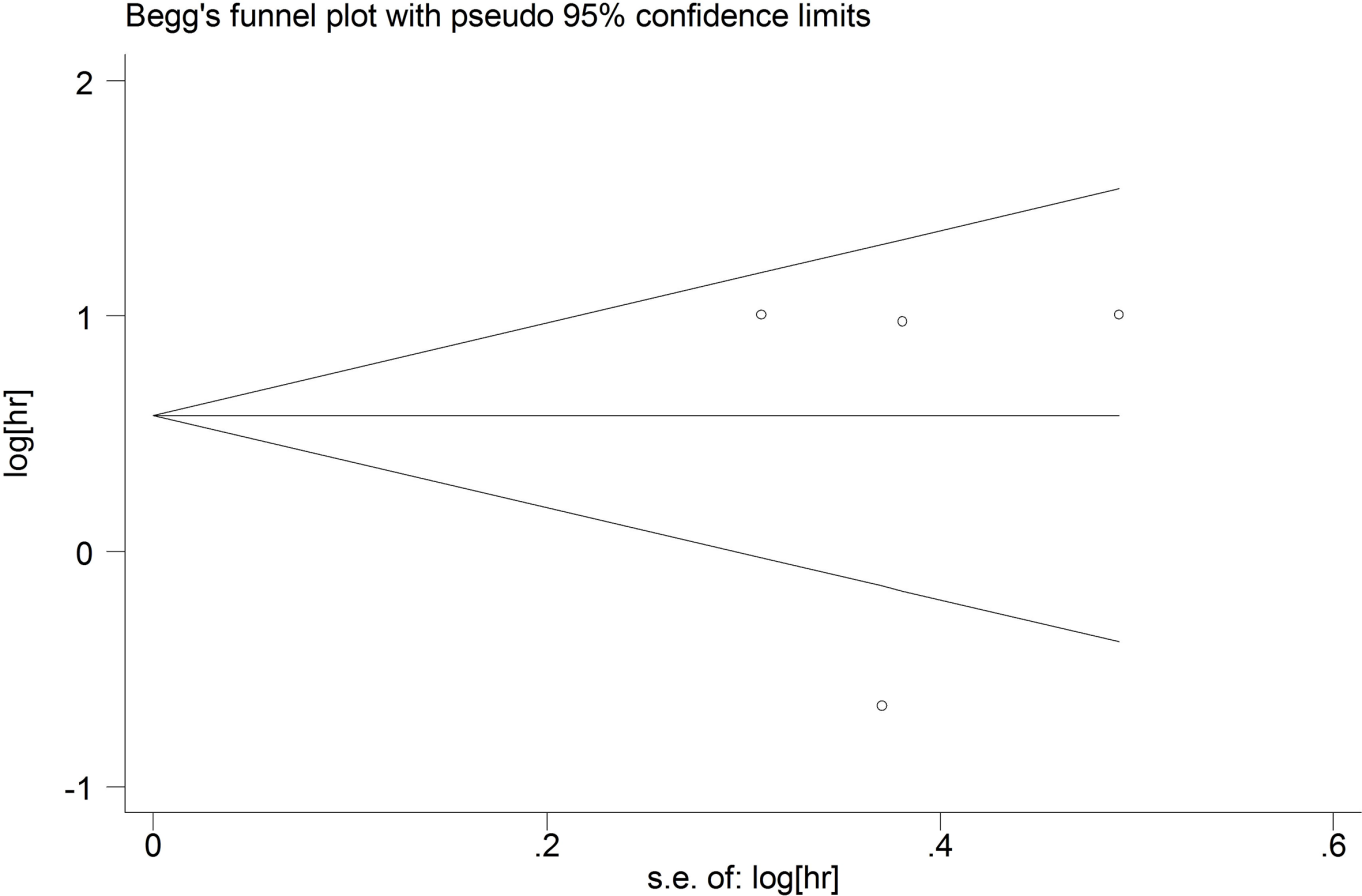
The publication bias for DFS/PFS/RFS.

We used funnel plots to qualitatively evaluate publication bias. Begg’s test and Egger’s test were used to quantitatively assess the publication bias. P values of Begg’s and Egger’s tests were 0.09 and 0.289 for OS (**Figure 7**), respectively. P values of Begg’s and Egger’s tests for DFS/PFS/RFS were 0.734 and 0.975, respectively (**Figure 8**). All P-values were more than 0.05, indicating that there was no publication bias.

## Discussion

Cancer is considered as the important obstacle to increase life expectancy worldwide (27). Promoting the early diagnosis and treatment of cancer is significant for the prognosis of patients with cancers. Therefore, it is important to find effective and appropriate prognostic markers. Nectin-4 as an important cellular adhesion molecule mediates intercellular and cell-cell matrix adhesion and cross-linking. Nectin-4 is considered as a tumor promoter (28).Cumulative studies have shown that nectin-4 is closely related to tumor prognosis, but no consensus has been reached. Therefore, we conducted the first meta-analysis to determine whether nectin-4 was a suitable prognostic marker in cancers.

Our meta-analysis included fifteen studies involving 2245 patients. The results showed that high nectin-4 expression was significantly associated with poorer OS. Subgroup analysis for OS showed that high nectin-4 expression mainly predicts poor prognosis in EC (HR: 1.78; 95% CI: 1.30–2.44) and GC (HR: 1.82; 95% CI: 1.50–2.22), suggesting that nectin-4 may have better predictive power for the two kinds of tumors. Our meta-analysis also revealed no association between high nectin-4 expression and DFS/PFS/RFS. We found that high nectin-4 expression significantly positively correlated with tumor diameter (big vs small), tumor stage (III-IV vs I-II) and infiltration depth (T3+T4 vs T2+T1). Moreover, we revealed that nectin-4 was closely associated with 12 proteins in BC. Taken together, these results indicated that nectin-4 may be a suitable and effective prognostic indicator for cancers.

Nectin-4 plays a crucial role in carcinogenesis and participates in tumor progression in different ways. Pavlova et al. showed that low expression of nectin-4 in BC could attenuate the adhesion between cancer cells and promote tumor cell apoptosis and distant metastasis through the β4/SHP-2/c-Src signaling pathway (29).Moreover, overexpression nectin-4 induced BC angiogenesis via endothelial integrin-beta4 (30). Nectin-4 could upregulate vascular endothelial growth factor (VEGF) expression to promote angiogenesis in PC (14). In LC, takano et al. revealed that interfering with nectin-4 expression could significantly inhibit tumor cell proliferation (16). They also found that exogenous nectin-4 expression increased the formation of lamellar lipoprotein and the invasive ability of mammalian cells by activating Rac1 signaling (16). In gallbladder and gastric cancers, nectin-4 promoted tumor cell proliferation, migration and invasion by activating PI3K/AKT signaling (20, 22). In colon cancer, overexpression nectin-4 significantly enhanced tumor cell proliferation, migration, colony formation and resistance to 5-fluorouracil (30).Mechanistically, nectin-4 may function in tumorigenesis by affecting afadin protein expression and the normal operation of the AKT-NF-κB signaling pathway (31).The epithelial-mesenchymal transition (EMT) is an important biological process in carcinogenesis (32). EMT is involved in the occurrence and development of numerous tumor types (33). Nectin-4 may promote tumor EMT by affecting a variety of adhesion proteins, including E-cadherin, N-cadherin and Vimentin (34, 35). The understanding of the specific mechanism of nectin-4 regulating tumors is insufficient. Single-cell technology and spatial transcriptomics can help us to further fully explore the role of nectin-4 in tumors (36, 37).

Although our study revealed that high nectin-4 expression could be a valuable prognostic biomarker in various cancers, some limitations need be noted. Firstly, all included studies had small sample sizes and were retrospective studies. Secondly, considering that there was certain level of heterogeneity, the results should be treated with caution. Thirdly, most of the included studies were from Asia. Furthermore, the research methods of different studies and cut-off values were inconsistent, which may affect the evaluation of nectin-4 as a prognostic biomarker. Finally, extracting HRs and 95% CIs from the survival curves may introduce slight errors.

Despite these limitations, our meta-analysis had some advantages. Firstly, this was the first meta-analysis to assess the association between nectin-4 expression and survival outcomes in different cancers. Secondly, we had identified potential causes of heterogeneity. Thirdly, sensitivity analysis revealed that the results were stable. Additionally, there was no obvious publication bias in this study.

In conclusion, we suggested that high nectin-4 expression predicted unfavorable OS. We also confirmed that nectin-4 can be applied as an effective prognostic indicator in cancers, especially for EC and GC.Nectin-4 may be a potentially important tumor target. More randomized controlled studies are required to validate our findings and further explore the association between nectin-4 and cancers.

## Data availability statement

The original contributions presented in the study are included in the article/supplementary material. Further inquiries can be directed to the corresponding authors.

## Author contributions

WW and ZQ contributed to the study inception and design. RL, KZ, and KW equally contributed to the literature search, analysis and writing of the manuscript. Other authors contributed to the study design and study supervision. All authors contributed to the article and approved the submitted version.
